# Redescription, complete mitochondrial genome and phylogenetic relationships of *Hexostoma thynni* (Delaroche, 1811) Rafinesque, 1815 (Monogenea, Hexostomatidae)

**DOI:** 10.1051/parasite/2022030

**Published:** 2022-05-23

**Authors:** Zouhour El Mouna Ayadi, Fadila Tazerouti, Romain Gastineau, Jean-Lou Justine

**Affiliations:** 1 Université des Sciences et de la Technologie Houari Boumediene, Faculté des Sciences Biologiques, Laboratoire de Biodiversité et Environnement : Interactions – Génomes (LBEIG) BP 32 El Alia, Bab Ezzouar Alger Algeria; 2 Institute of Marine and Environmental Sciences, University of Szczecin Szczecin Poland; 3 ISYEB, Institut de Systématique, Évolution, Biodiversité (UMR7205 CNRS, EPHE, MNHN, UPMC, Université des Antilles), Muséum National d’Histoire Naturelle CP 51 55 rue Buffon 75231 Paris Cedex 05 France

**Keywords:** Monogenea, Mitogenome, Phylogeny, Systematics, Hexostomatidae

## Abstract

Specimens of *Hexostoma thynni* (Delaroche, 1811) Rafinesque, 1815 were collected from their type-host, the bluefin tuna *Thunnus thynnus*, caught off Algeria, i.e. close to the type-locality, off Mallorca, which is also in the Mediterranean. The species is briefly redescribed and compared to previous descriptions, under the same name or as its synonym *Plagiopeltis duplicata* Diesing, 1858, to ascertain identity of specimens. The three genera within the Hexostomatidae (*Hexostoma* Rafinesque, 1815, *Neohexostoma* Price, 1961 and *Homostoma* Unnithan, 1965) are briefly discussed, with comments on the fragility of characters used to distinguish them. Using next-generation sequencing, the complete mitogenome and the cluster of ribosomal genes (SSU, LSU, ITS1, ITS2, 5.8S) were obtained. The mitogenome is 14,649 bp long and codes for 12 protein-coding genes, 2 ribosomal RNA genes and 22 transfer RNA genes; its size is similar to other mitogenomes obtained from polyopisthocotylean monogeneans. A phylogeny based on concatenated mitogenome protein-coding genes from nine species of polyopisthocotylean monogeneans produced a tree in which the Hexostomatidae *H. thynni* was associated with other Mazocraeidea, such as Chauhaneidae and Diclidophoridae. This invalidates the hypothesis of Boeger & Kritsky (1993) of Hexostomatidae as sister-group to the Mazocraeidea and suggests the demise of the suborder Hexostomatinea Boeger & Kritsky, 1993. We insist on the usefulness of depositing parts of specimens used for molecular analyses, prepared on permanent slides, in a curated collection.

## Abstract (fr)

Des spécimens d’*Hexostoma thynni* (Delaroche, 1811) Rafinesque, 1815 ont été collectés sur leur hôte-type, le thon rouge *Thunnus thynnus*, capturé au large de l’Algérie, c’est-à-dire près de la localité-type, au large de Majorque, qui se trouve également en Méditerranée. L’espèce est brièvement redécrite et comparée aux descriptions précédentes, sous le même nom ou sous son synonyme *Plagiopeltis duplicata* Diesing, 1858, pour vérifier l’identité des spécimens. Les trois genres au sein des Hexostomatidae (*Hexostoma* Rafinesque, 1815, *Neohexostoma* Price, 1961 et *Homostoma* Unnithan, 1965) sont brièvement discutés, avec des commentaires sur la fragilité des caractères utilisés pour les distinguer. En utilisant le séquençage de nouvelle génération, le mitogénome complet et le groupe de gènes ribosomiques (SSU, LSU, ITS1, ITS2, 5.8S) ont été obtenus. Le mitogénome a une longueur de 14 649 pb et code pour 12 gènes codant pour des protéines, 2 gènes d’ARN ribosomal et 22 gènes d’ARN de transfert, et sa taille est similaire à celle des autres mitogénomes obtenus de monogènes Polyopisthocotylea. Une phylogénie basée sur les gènes codant pour les protéines concaténées du mitogénome de 9 espèces de monogènes Polyopisthocotylea a produit un arbre dans lequel l’Hexostomatidae *H. thynni* était associé à d’autres Mazocraeidea tels que les Chauhaneidae et les Diclidophoridae. Ceci réfute l’hypothèse de Boeger & Kritsky (1993) des Hexostomatidae comme groupe-frère des Mazocraeidea et suggère la disparition du sous-ordre Hexostomatinea Boeger & Kritsky, 1993. Nous insistons sur l’intérêt de déposer dans une collection entretenue des parties des spécimens utilisées pour les analyses moléculaires, préparées sur des lames microscopiques permanentes.

## Introduction

Monogeneans are fascinating parasites, showing a wide variety of morphologies and habitats on their hosts. In the last few decades, various attempts have been made to elucidate their phylogenetic relationships, based on characters such as spermatozoa [32], morphology [9, 10] and various DNA sequences [31, 42, 43, 45, 46]. DNA sequences were generally based on relatively short sequences of ribosomal DNA such as partial 18S genes, partial 28S genes, or both. On the other hand, partial sequences of mitochondrial cox1 (or COI) have demonstrated excellent potential for discriminating species [3–5, 12–15, 19, 20]. Next-generation sequencing now allows us to obtain the whole cluster of nuclear ribosomal DNA and the complete mitogenome, increasing the length of available sequences by an order of magnitude, and therefore providing more information for phylogeny. We present here a study concerning *Hexostoma thynni* (Delaroche, 1811) Rafinesque, 1815, a species that has a large (1–2 cm) and thick body, making it simple to extract sufficient quantities of DNA for sequencing.

## Material and methods

### Fish

From January to April 2021, 20 specimens of *Thunnus thynnus* were purchased dead from fishermen. The fish had been caught off the Algerian coast near Bordj El Bahri (36° 47′ 26″ N, 3° 14′ 59″ E). Fish were identified using keys [27]. Gills were extracted from each fish, stored in a plastic box and examined in the laboratory for monogeneans. The specific identity of one host-fish was confirmed by molecular analysis, with a sequence extracted from the blood which was associated with the sequenced monogenean specimen (Table 1).

**Table 1:** Specimens of *Hexostoma thynni*: vouchers for molecular studies. Monogeneans were collected from two fish specimens. Fish ThyBr01 provided two monogenean specimens. Fish ThyBr02 provided five monogenean specimens. For each monogenean specimen, the slide includes the anterior and posterior extremity (stained and mounted in Canada balsam) and the vial contains the midbody. In addition, 10 whole monogenean specimens were mounted on permanent slides and are deposited as MNHN HEL1806–HEL1815.

Fish ID	Monogenea id	Slide	Vial	Molecular information
*Thunnus thynnus*	*Hexostoma thynni*			
ThyBr01			Fish gill	
	Thy01Mo1	HEL1801	Midbody destroyed	
	Thy01Mo2		HEL1799	
ThyBr02			Fish gill	Information extracted from blood in digestive tract of monogenean specimen Thy02Mo1
	Thy02Mo1	HEL1757	Midbody destroyed	Complete mitogenome, GenBank OM764630; Ribosomal Cluster, GenBank OM731590
	Thy02Mo2	HEL1802	HEL1802	
	Thy02Mo3	HEL1803	HEL1803	
	Thy02Mo4	HEL1804	HEL1804	
	Thy02Mo5	HEL1805	HEL1805	

### Monogeneans

**Figure 1:**
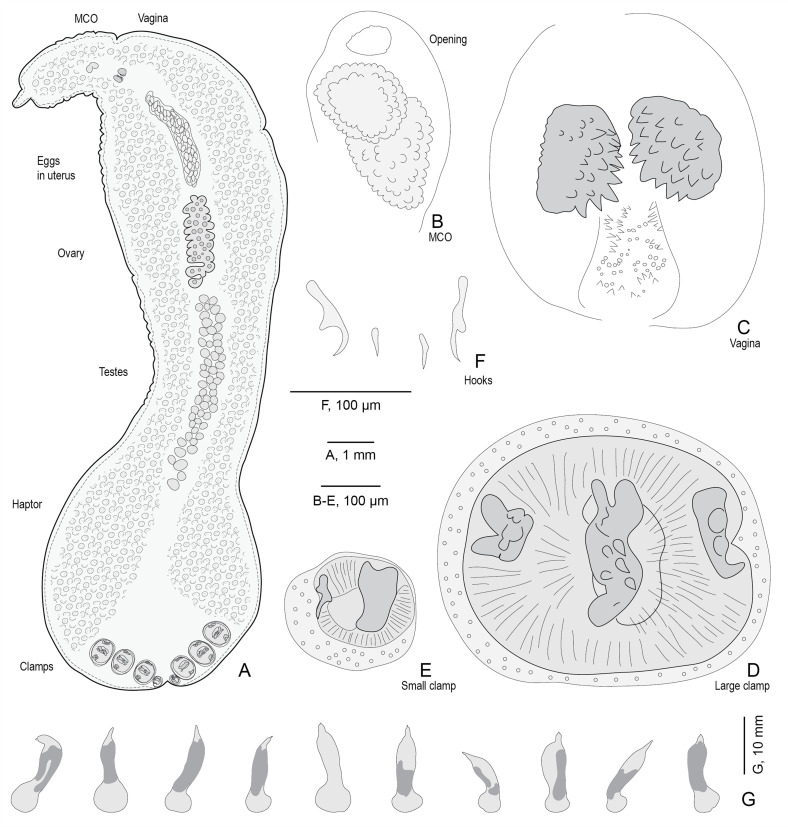
Drawings of specimens of *Hexostoma thynni* (Delaroche, 1811) Rafinesque, 1815 from *Thunnus thynnus* collected off Algeria. A, whole body; not all testes are figured; intestinal diverticula not figured, but colocalized with vitellarium. B, male copulatory organ. C, vagina. D, large clamp. E, small clamp. F, hooks. G, ten specimens on slides, showing only the silhouette and the dark zone within the body. A–F, slide MNHN HEL1806; G, slides MNHN HEL1806-1815.

**Figure 2:**
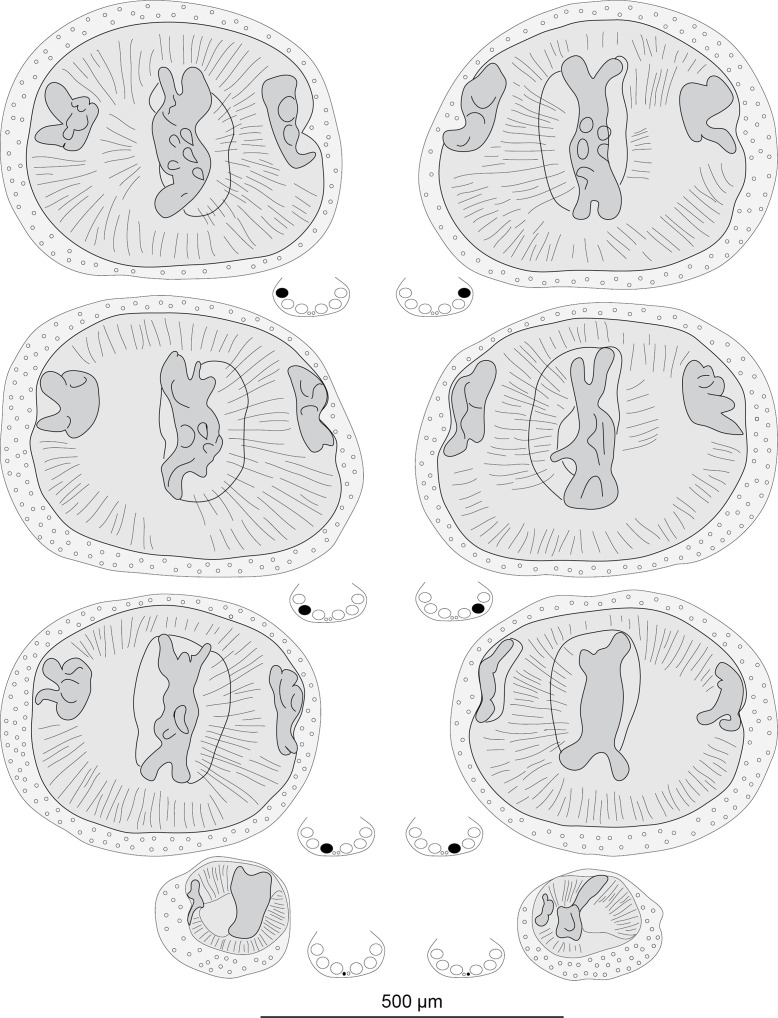
All clamps of a specimen of *Hexostoma thynni*. The sketches indicate the position of each clamp. Slide MNHN HEL1806.

Monogeneans (Table 1) were removed and preserved in 70% ethanol, stained with acetic carmine, dehydrated in ethanol series (70%, 96% and 100%), cleared in clove oil and finally mounted in Canada balsam. Drawings were made with an Olympus BH2 microscope with drawing tube. Drawings were scanned and redrawn on a computer using Adobe Illustrator (Figs. 1 and 2). Measurements were made with a Carl Zeiss Axioplan microscope and are in micrometers (μm). Measurements are indicated as the mean, followed by the range and number of measurements in parentheses. For the silhouette of bodies (Fig. 1G), the slides were placed in a standard office photocopying machine, scanned at 600 ppm, and the monogeneans were drawn with Adobe Illustrator, showing only the silhouette and the dark zones within the body.

### Sequencing

**Figure 3:**
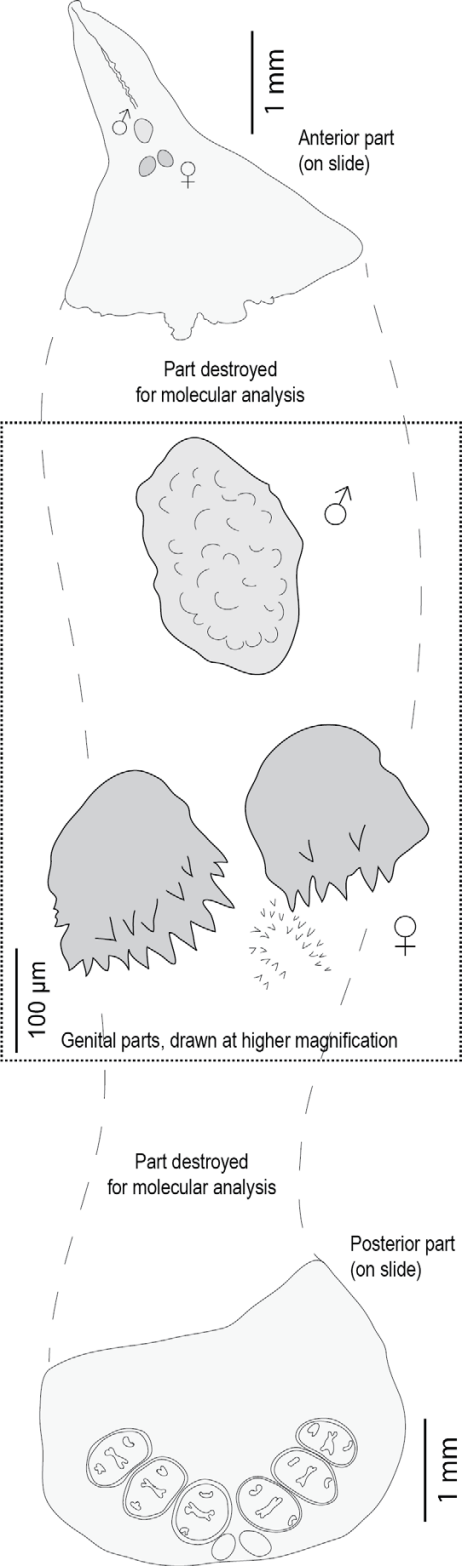
Specimen of *Hexostoma thynni* used for the molecular study. The anterior and posterior parts were mounted on a slide (MNHN HEL1757); the silhouette of the middle part, destroyed for analysis, is shown as dotted lines. The genital organs, from the anterior part, are drawn at a higher magnification in the middle.

A specimen (MNHN HEL1757) was cut into three parts; the anterior and posterior parts were prepared on a permanent slide and drawn (Fig. 3) to ensure that this specimen was conspecific with other specimens from the same host; the middle part was stored in 98% ethanol. The part stored in ethanol was sent to the Beijing Genomics Institute in Shenzhen, China, where DNA was extracted according to their internal protocol. Sequencing was performed on a DNBSEQ platform. A total of ca. 40 million clean 150 bp paired-end reads was obtained. Reads were assembled using SPAdes 3.14.0 [6] with a k-mer of 125. The mitogenome was retrieved from the contig file, circularized and trimmed. Annotation was performed with the help of MITOS [7]. The map of the mitogenome was drawn with OGDRAW [39]. The contig corresponding to the cluster of nuclear ribosomal RNA genes was also retrieved from the contig file. The positions of the rRNA genes and ITS were found with the help of Rfam [35].

For characterization of fish DNA from the gut of the monogenean, data mining was performed on the contigs obtained after assembly to find potential traces of alien DNA, using blastn command line [11] and a database consisting of the complete mitogenome of *T*. *thynnus* obtained from GenBank (accession number: GU256522). No e-value filter was employed for the blastn query.

### Phylogeny

All the protein-coding genes sequences were extracted from the mitogenome of *H. thynni* and nine other species of Polyopisthocotylea. For each species, amino-acid sequences of the mitochondrial proteins were concatenated by alphabetic order. Then, concatenated sequences were aligned using MAFFT 7 [36] with the -auto option. The alignment was later automatically trimmed using trimAl [18] with the -automated1 option, resulting in a standard size of 3338 AA for each species. A maximum likelihood (ML) phylogeny was generated using RaxML 8.0 [59]. The best evolutionary model was chosen using ModelTest-NG v0.1.7 [22] based on the Bayesian information criterion (BIC), which returned mtZOA + G4 + F as the best model. The best tree out of 100 was computed for 1000 bootstrap replicates.

## Results

### Brief redescription of *Hexostoma thynni* (Figs. 1–3)

Host: *Thunnus thynnus* (Linnaeus, 1758), Atlantic bluefin tuna

Locality: Off Bordj El Bahri, Algerian coast, Mediterranean Sea

Site on host: Gills

Prevalence: 30%

Based on 10 specimens on slides (MNHN HEL1806-1815).

The internal anatomy was hard to see in most specimens, because of the thickness of the body and the presence of heavy deposits of blood remnants in the digestive system. In addition, the only sclerotized parts of the genital system are in the vagina and their size is small compared to body size.

Silhouettes of our 10 specimens on permanent slides are shown on Figure 1G, in an attempt to evaluate the character of “waist-like constriction of the body” used by Yamaguti (1963) [64] and show the extent of blood in the intestine; see Discussion.

Measurements are mentioned in Table 2. Body elongate (Fig. 1A), length of body proper including haptor 14,080 (12,000–15,000, *n* = 10). Maximum width at level of ovary 2750 (2000–4000, *n* = 10). Haptor not delimited from body proper, 4300 (3000–6000, *n* = 10) long and 3300 (2000–5000, *n* = 10) wide. Haptor armed with four pairs of clamps organized in two transversal rows and two hooks placed posteriorly. Each clamp provided with one middle sclerite (Fig. 1D), 238 (217–275, *n* = 10) in length and two lateral sclerites, 120 (93–149, *n* = 10) in length. Anteriormost pair of clamps 532 (456–626, *n* = 10) long, 430 (340–522, *n* = 10) wide, second pair 572 (488–674, *n* = 10) long, 428 (392–455, *n* = 10) wide, third pair 534 (453–591, *n* = 10) long, 467 (414–565, *n* = 10) wide, most posterior pair much smaller than the three first pairs (Fig. 1E) 211 (164–266, *n* = 9) long, 149 (111–173, *n* = 9) wide. Hooks hard to distinguish (Fig. 1D), small hooks 24 (23–25, *n* = 3) long, large hooks 88 (59–103, *n* = 5) long.

**Table 2:** Measurements of *Hexostoma thynni* (μm), designated under the name *Hexostoma thynni* or *Plagiopeltis duplicata*. We consider that specimens described by Parona & Perugia (1892) and Palombi (1943) correspond to *H. thynni*, such as our own specimens, but that the specimens measured by Lopez-Roman & De Armas Hernandez (1989), which are much larger, probably correspond to another species.

Name used	*Hexostoma thynni*	*Plagiopeltis duplicata*	*Hexostoma thynni*	*Hexostoma thynni*
Hosts	*Thunnus thynnus*	*Thunnus thynnus*	*Thunnus thynnus, Sarda sarda*	*Thunnus obesus, Thunnus thynnus*
Source	Present study	Parona & Perugia (1892) [50]	Palombi (1943) [47]	Lopez-Roman & De Armas Hernandez (1989) [40]
Localities	Off Algeria, Mediterranean	Off Italy, Mediterranean	Off Italy, Mediterranean	Off Canary Islands, Atlantic Ocean
Number of specimens	10	?	?	35
Body length	14080 (12000–15000)	14000–16000	14000–16000	19120–31530
Body width	2750 (2000–4000)			4240–7520
Haptor length	4300 (3000–6000)			3100–6700
Haptor width	3300 (2000–5000)	6000		
Clamps, length × width	1st pair: 532 (456–626) × 430 (340–522)	External clamps: 500–700	Anterior clamps: 700 × 500	Large clamps: 500–1000 × 360–660
	2nd pair: 572 (488–674) × 428 (392–455)			
	3rd pair: 534 (453–591) × 467 (414–565)			
	4th pair: 211 (164–266) × 149 (111–173)	Small clamps: 320 × 190	Posterior clamps: 320 × 190	Small clamps: 218–473 × 146–318
Marginal and × sclerite of 1st clamp	120 (93–149), 238 (217–275)			
Marginal and × sclerite of 2nd clamp	141 (112–164), 248 (232–284)			
Marginal and × sclerite of 3nd clamp	138 (107–161), 234 (215–249)			
Marginal and × sclerite of 4th clamp	65 (59–77), 96 (77–111)			
Posterior anchor	24 (23–25)	23	25	18–29
Anterior anchor	88 (59–103)	91	100	70–122
Buccal organ, length × width	41 (34–47) × 28 (25–31)		90 × 45	
Pharynx, length × width	73 (69–76) × 51 (46–54)			
Genital atrium to anterior extremity	1466 (1040–1671)			
Genital atrium length	249 (196–308)			
Genital atrium width	147 (119–175)			
Number of testes	71 (64–75)			86–112
Vagina to anterior extremity distance	2040 (1584–2426)			
Vaginal sclerite, length × width	197 (172–281) × 137 (127–150)	Length 160–230	200 (length)	130–300 × 109–273
Egg, length × width	269 (228-390) × 147 (100–265)	229 × 120	230 × 120	
Egg filament length	239 (179–297)		15	

Pharynx oval, 73 (69–76, *n* = 5) long, 51 (46–54, *n* = 5) wide. Oral suckers not seen. Esophagus bifurcating into two intestinal ceca with numerous diverticula, not united posteriorly, extending into haptor. Intestinal ceca often filled with blood or remnants of digested blood, producing dark zones in body, highly variable depending on the specimen; see silhouettes of 10 specimens in Figure 1G.

Genital atrium located at 1466 (1040–1671, *n* = 9) from anterior end of body. Male copulatory organ unarmed, a highly folded structure (Fig. 1B), 249 (196–308, *n* = 8) long, 147 (119–175, *n* = 8) wide. Tests rounded, posterior to ovary, hard to count with precision, 71 (54–75, *n* = 6) in number, 188 (132–225, *n* = 9) long, 162 (121–212, *n* = 9) wide. Vas deferens sinuous passes through midline of body from testis to genital atrium.

Ovary pretesticular, sinuous and very contorted. Vagina at 2040 (1584–2426, *n* = 10) from anterior end of body and armed with two dorsal hemispherical bodies, 197 (172–281, *n* = 9) long, 137 (127–150, *n* = 4) wide, each provided with sawtooth-like spines directed towards centre (Fig. 1C); group of small spines located posteriorly and ventrally to hemispherical bodies. Uterus median and ventral containing numerous eggs. Vitellarium very developed with numerous follicles occupying whole body except central zone; vitellarium generally colocalized with intestinal diverticula. Eggs, ovoid (Fig. 1G), 269 (228–390, *n* = 5) long and 147 (100–265, *n* = 5) wide with two filaments; filaments 239 (179–297, *n* = 4) long.

### The mitogenome

**Figure 4:**
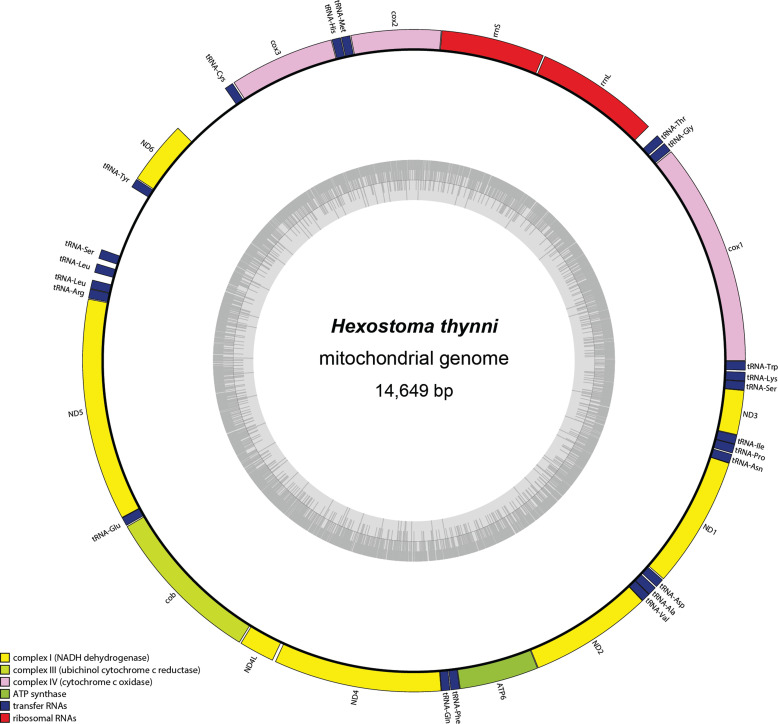
Complete mitogenome of *Hexostoma thynni*, specimen MNHN HEL1757 from off Algeria. The mitogenome is 14,649 bp long and codes for 12 protein-coding genes, 2 ribosomal RNA genes, and 22 transfer RNA genes.

The mitogenome is 14,649 bp long (GenBank accession number: OM764630). It codes for 12 protein-coding genes, 2 ribosomal RNA genes and 22 transfer RNA genes (Fig. 4). All genes are coded on the same strand. The genome has two long non-coding regions, between tRNA-Cys and *ND6* (434 bp) and between tRNA-Tyr and tRNA-Ser (489 bp), also located close to *ND6*. A premature stop followed by a tRNA was found for three genes, namely *cox2*, *ND2* and *ND5*. The size of the mitogenome is compared with those reported for other Polyopisthocotylea in Table 3.

**Table 3:** Mitogenomes of polyopisthocotylean monogeneans. The table indicates the size of the mitogenomes and the precautions taken by authors to ascertain the systematics of the specimens they used.

Species	GenBank accession number	Size of the mitogenome	Systematics of specimens	Reference
*Eudiplozoon* sp.	MG458328	14,334 bp	No species identification, images as supplementary files	[66]
*Heterobothrium okamotoi*	MK948930	14,642 bp	Similar specimen deposited	[37]
*Hexostoma thynni*	OM764630	14,649 bp	Specimen used for sequencing drawn and deposited	Present paper
*Microcotyle caudata*	MT180126	14,267 bp	Similar specimen deposited	[44]
*Microcotyle sebastis*	DQ412044	14,407 bp	No specimen	[49]
*Paradiplozoon opsariichthydis*	MG458327	15,385 bp	Images as supplementary files	[66]
*Polylabris halichoeres*	JF505509	15,527 bp	No specimen	[68]
*Pseudochauhanea macrorchis*	JN592039	15,031 bp	No specimen	[67]
*Sindiplozoon* sp.	MG458326	15,254 bp	No species identification, images as supplementary files	[66]

### The cluster of nuclear RNA

We obtained a 7855 bp sequence that seems to comprise the complete cluster of nuclear rRNA, distributed as follows: 2002 bp (18S), 1007 bp (ITS1), 158 bp (5.8S), 551 bp (ITS2) and 4141 bp (28S). Prior to this study, GenBank contained eight sequences belonging to *H. thynni*, all corresponding to partial 28S or ITS2 and their neighbouring regions. An alignment of our sequence with those corresponding to ITS (EF653393, EF653392, EF653391, EF653390) showed that all sequences were identical. Partial 28S sequences were identical between them, except one (EF653381) which had one nucleotide difference with the others (EF653382, EF653383 and our own material).

### Phylogeny based on mitogenome protein-coding genes

**Figure 5:**
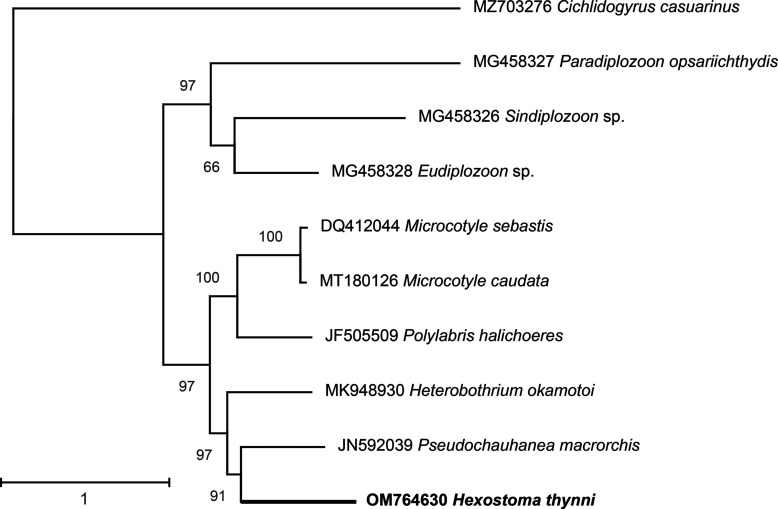
Phylogenetic tree based on protein-coding genes sequences of nine species of Polyopisthocotylea, with the Monopisthocotylea *Cichlidogyrus casuarinus* as outgroup. The tree has two large clades. The first one contains only the Diplozoidae. The second includes members of the Mazocraeidea and is subdivided into two smaller subclades, with one containing the Microcotylidae and the other containing the Chauhaneidae, Diclidophoridae and Hexostomatidae (*H. thynni*). *Hexostoma thynni* is not the sister-group to the other Mazocraeidea.

The Monopisthocotylea *Cichlidogyrus casuarinus* was used as an outgroup. The tree (Fig. 5) had two large clades. The first one contained only the Diplozoidae (*Eudiplozoon* sp., *Paradiplozoon opsariichthydis*, *Sindiplozoon* sp.). The second included members of the Mazocraeidea and was subdivided into two smaller subclades, with one containing the Microcotylidae (*Polylabris halichoeres*, *Microcotyle* spp.), the other containing the Chauhaneidae (*Pseudochauhanea macrorchis*), Diclidophoridae (*Heterobothrium okamotoi*) and Hexostomatidae (*H. thynni*). The less supported node was the one associating *Sindiplozoon* sp. and *Eudiplozoon* sp., with support of 66%, but all the nodes supports in the cluster containing Chauhaneidae, Diclidophoridae and Hexostomatidae ranged from 91% to 100%. The tree associated *H. thynni* and *P. macrorchis* with a support of 91%, i.e. the Hexostomatidae were not the sister-group to the Mazocraeidea.

### Host DNA and identification

We obtained a blastn match from the fish blood within the gut of the monogenean, which consisted in a fragment of the NADH dehydrogenase subunit 4 (*ND4*) gene of *T*. *thynnus.* The fragment was 288 bp long and was assembled with a low coverage of 0.92×. The fragment was submitted to an online megablast query. The best results were, with the same percentage of identity of 99.65%, the various mitogenomes of *T. thynnus* with accession numbers KF906720, MT410869, JN086149, GU256522 and AB097669. In all cases, there was a single, identical polymorphism (G for the reference genomes and A for the gut DNA). From the information available on GenBank, none of these mitogenomes seem to have been obtained from tunas from the Mediterranean Sea. The polymorphism is a silent mutation that does not change the predicted amino-acid sequence of this part of the ND4 protein; however, due to the low coverage, the validity of this polymorphism should be taken with care. In any case, this confirms the identity of the host with molecular tools.

## Discussion

### Historical account of *Hexostoma thynni*

*Hexostoma thynni*, the type-species of the genus, was originally described (as *Polystoma thynni* Delaroche, 1811) from the gills of *T. thynnus* (designated as *Scomber thynnus* Linnaeus) from specimens collected off Mallorca, in the Mediterranean Sea [23]. The original description by Delaroche is short and amusingly erroneous in the orientation of the body, with the large clamps considered as anterior and being six minute mouths [23]; however, this description clearly sets the type-host and the type-locality for the species. This paper provides an opportunity to remark that several subsequent authors curiously used typographic variation for Delaroche’s name (such as “De La Roche” or “La Roche”); however, the original description is clearly authored Delaroche.

Rafinesque (1815) placed the species in his new genus *Hexostoma* Rafinesque, 1815 [54]; although the etymology is not explained, it is clear that it corresponds to Delaroche’s interpretation, as *Hexo*- (Greek: six) and –*stoma* (Greek: mouth). Blainville (1828) examined Delaroche’s specimens, corrected his mistakes and correctly interpreted the six clamps, and therefore “changed the name of the genus” to *Hexacotyla* Blainville, 1828 [8]. Rudolphi (1819) placed the species in the genus *Polystoma* like Delaroche but changed the species name to *Polystoma duplicatum* Rudolphi, 1819 [57].

Diesing (1858) provided a description of *Plagiopeltis duplicata* Diesing, 1858, including a color plate showing the body and one clamp [24]; the description was based on one specimen collected in 1836 from *Sarda sarda* (designated as *Thunnus brachypterus*) off the Balearic Islands, Mediterranean Sea, and kept in the Imperial Collection of Vienna. Diesing clearly indicated that this was the same species as *Polystoma thynni* but nevertheless proposed a new name. Parona & Perugia (1892) redescribed *P. duplicata* from specimens collected from *Sarda sarda* (designated as *Pelamis sarda*) collected off Genoa, Italy (Mediterranean Sea) [50]. They considered that their specimen was very similar to the illustration given by Diesing (1858), especially with eight suckers including two central ones that are smaller. They provided a detailed description and a plate showing the general shape of the body, with a precise drawing of the intestinal diverticula reaching all parts of the body; indeed, Hayward (1985) [29] confirmed later that, in Hexostomatidae, small lateral branches along the ceca fuse to form “an extensive reticulated net”. Parona & Perugia (1892) also provided a description of the male and female copulatory parts. In their Figure 12, Plate 3, the male copulatory organ is figured as an evaginated structure with spines; we agree that the internal folded structure in our specimens might be able to evaginate, but we did not notice spines. Measurements provided by Parona & Perugia are shown in our Table 2.

*Hexostoma thynni* was redescribed by Palombi (1943) from specimens collected from *T. thynnus*, its type-host, and *Sarda sarda* (Bloch), from various localities off Italy, again in the Mediterranean Sea [47]. The illustration included the general shape of body, clamps, eggs and haptoral hooks, but no internal anatomy. Curiously, most measurements reported by Palombi (1943) [47] are identical to those published by Parona & Perugia in 1892 [50] (see our Table 2). Palombi (1949) [48] reproduced some of the drawings from his 1943 paper. Euzet (1955) described the oncomiracidium [25] but not the adult. Lopez-Roman & De Armas Hernandez (1989) provided a brief unillustrated redescription of *H. thynni* from *T. obesus* and *T. thynnus* off Tenerife Island (Atlantic Ocean) [40]; their measurements, shown in our Table 2, are those of specimens much larger than the others, suggesting that it was not the same species.

In spite of a search in the monogenean literature, we did not find any drawings of *H. thynni* that are more recent, or more detailed, than those of Palombi [47] (however, see below for *H. lintoni* Price, 1961).

In application of the Principle of Priority, *Hexostoma* is valid and *Hexacotyla* is only a synonym, and *Plagiopeltis duplicata* is a junior synonym of *H. thynni*. We therefore conclude that *Hexostoma thynni* (Delaroche, 1811) Rafinesque, 1815 is the valid binomial and that *Polystoma thynni* Delaroche, 1811, *Polystoma duplicatum* Rudolphi, 1819, *Hexacotyla thynni* (Delaroche, 1811) Blainville, 1828 and *Plagiopeltis duplicata* Diesing, 1858 are its synonyms.

### Identification of our specimens as *Hexostoma thynni*

Within *Hexostoma*, eight species are currently considered valid in WoRMS [62]. Our specimens have in common with previous descriptions of *H. thynni* the type-host and the locality in the Mediterranean Sea. Table 2 shows that measurements are not different from those reported by Palombi (1943) [47] and Parona & Perugia (1892) [50]. We are therefore confident that our specimens belong to *H. thynni*.

*Hexostoma thynni* has been recorded several times in the Mediterranean on different hosts, mainly its type-host *T. thynnus* [2, 21, 26, 41, 53, 60] and also *Katsuwonus pelamis* (Linnaeus) [60], *S. sarda* (Bloch) [47], *Thunnus obesus* (Lowe) and *Auxis thazard* (Lacepède) [16]. It has also been recorded from *T. thynnus* in the North Atlantic Ocean [55] and from *Thunnus albacares* (Bonnaterre) and *T. maccoyii* (Castelnau) off Australia [2, 30]. As is the case for many polyopisthocotylean species for which the original descriptions lack details, it is likely that most of these records, apparently precise at the specific level, were done mostly on the basis of the host and rapid identification of the basic family characters of the monogenean. The effect of *H. thynni* on the pathomorphology of gills of *T. maccoyii* was studied by Adams et al. (2017 [1]; curiously, only six clamps are mentioned in the text and shown on one illustration.

Linton mentioned *H. thynni* from *S. sarda* off Massachusetts, USA [38]. Price, from Linton’s single specimen which he considered himself in bad condition and which “did not stain well”, erected the species *Hexostoma lintoni* Price, 1961 [52]. We are not convinced by the differences presented for differentiating *H. lintoni*, which is likely a junior synonym of *H. thynni*. However, we do not propose a formal nomenclatural change without examining the specimen.

### Considerations on the Hexostomatidae

The Hexostomatidae Price, 1936 are mainly differentiated from other polyopisthocotylean monogeneans by the structure of their clamps, defined by Price (1961) as: “sucker-like clamps containing three dissimilar sclerites, two small, irregular and tending to be bipartite, one on either side of lateral wall of capsule, and a larger, more or less saddle-shaped sclerite in middle” [52]. The vagina contains “a pair of opposing hemispherical bodies armed on their free margins with backwardly directed spines” [52]. Indeed, the clamps of the hexostomatid are clearly different from that of all polyopisthocotyleans and are marked by the reduction of sclerites [58].

The Hexostomatidae include three genera in WoRMS [63]: *Hexostoma* Rafinesque, 1815, *Neohexostoma* Price, 1961, and *Homostoma* Unnithan, 1965 [52, 54, 61]; most species parasitize the gills of scombrids [52]. *Unnithania* Gupta & Sachdeva, 1988, proposed in an abstract [28] for *Unnithania indica* Gupta & Sachdeva, 1988, is considered “dubious” in WoRMS [63]. The host of this species is a carangid, not a scombrid, no detail was given on the structure of the clamps, and the description of the genital apparatus is widely different from hexostomatids [28]: for these reasons, we even doubt that the species was a hexostomatid and we do not consider it further.

The three genera *Hexostoma*, *Neohexostoma* and *Homostoma* are distinguished on characters of clamps and general shape of body. *Neohexostoma* was proposed by Price (1961) and distinguished from *Hexostoma* on the following characters: posterior end of body not truncate (vs. truncate in *Hexostoma*) and clamps in two more or less vertical rows (vs. clamps in a more or less straight row in *Hexostoma*) [52]. It was also indicated that the posterior clamps were only slightly smaller than the three anterior ones (vs. posterior (or innermost) clamps much smaller than others in *Hexostoma*) [52]. Yamaguti (1963) accepted *Neohexostoma* but distinguished it from *Hexostoma* on the characters of “an elongate waist-like constriction present anterior to opisthophaptor, clamps in two more less vertical rows”, vs. “no waist-like constriction between testicular region and opisthophaptor and clamps in two more or less vertical rows in *Hexostoma*” [64]. Unnithan (1965) proposed *Homostoma*, writing “unlike *Hexostoma*, […] the haptor is tongue-shaped or triangular and demarcated from the body by deep lateral constrictions”; the four pairs of clamps were of identical sizes [61]. Rohde (1978) examined several type specimens; he did not mention *Homostoma*, but concluded “the absence of any significant and consistent differences between *Hexostoma* and *Neohexostoma* and the great similarity of some species with regard to the structure of clamps and the genital system indicate that *Neohexostoma* must be considered a synonym of *Hexostoma*” [56]. Our Figure 1G, showing the silhouette of 10 specimens of *H. thynni*, shows that Yamaguti’s character of “waist-like constriction” might be hard to use, especially when descriptions are based on a few specimens. From a general point of view, characters based on soft parts of the body, which are subject to change according to fixation and pressure during preparation of slides, are unreliable in monogeneans.

If we dismiss the character of body shape as subjective and ill-characterized, the three genera can be distinguished only on the basis of the size of the fourth clamp pair compared to the three others, with the fourth pair much smaller (*Hexostoma*), slightly smaller (*Neohexostoma*) or equal (*Homostoma*). Again, the limits between “slightly smaller” and “equal” appear somewhat subjective, with the example of *Hexostoma kawakawa* Yamaguti, 1968, in which the drawing shows subequal clamps on one side and a slightly smaller posterior clamp on the other side [65]. However, Zhu et al. (2020) proposed a key to species of *Neohexostoma* [69].

Finally, we consider that the three genera *Hexostoma*, *Neohexostoma* and *Homostoma* require a detailed evaluation, which is not the scope of the present paper, and we provisionally consider them valid; we expect, however, that the abundant molecular information that we provide here on the type species of *Hexostoma*, itself the type-genus of the Hexostomatidae, will allow detailed comparative molecular studies in the future.

### New sequences for the identification of *Hexostoma thynni*

Prior to this study, GenBank did not contain any mitochondrial sequences belonging to a species of the genus *Hexostoma*, or to a broader extent, to the Hexostomatidae family. Availability of molecular markers started at the order level, and the same situation happened for the widely used small subunit of the nuclear ribosomal RNA genes (SSU or 18S). In this paper, we provide for the first time mitochondrial sequences for this species, and its complete mitochondrial genome. We also provide the complete cluster of nuclear ribosomal RNA genes, for which only ITS2 and very partial 28S were available until now for this species. We believe that our study, based on morphological identification and redescription from the type-host caught very close to the type-locality, provides robust molecular information for the species, and comparative material for other monogeneans.

### Molecular phylogeny and consequences for the phylogenetic position of the Hexostomatidae

With only nine complete mitogenomes used for the phylogeny of the Polyopisthocotylea, we certainly cannot redefine the phylogeny of this group. However, the tree based on concatenated protein genes separated the diplozoids (parasites of freshwater fishes) from the Mazocraeidea (all parasites of marine fish for the species studied here). The three microcotylids were grouped together, which is certainly not a surprising result, and the three other species each belonged to a different family (diclidophorids, chauhaneids and hexostomatids). An important result, however, is that the hexostomatid was not the sister-group to the members of the Mazocraeidea, but was rather a branch within members of this order. Bychowsky (1957) included the Hexostomatidae within the Mazocraeidea [17], but Boeger & Kritsky (1993) proposed a new suborder Hexostomatinea Boeger & Kritsky, 1993 and their Figure 16 clearly showed the Hexostomatidae as a sister-group to all other Mazocraeidea [9]. Although this is of minor importance in comparison to our new molecular results, we note that sperm ultrastructure was not different in *Hexostoma* sp. from that of other Polyopisthocotylea [33]. Our molecular study invalidates the hypothesis of Boeger & Kritsky; in terms of high-level systematics, the Hexostomatinea are demised and the Hexostomatidae are members of the Mazocraeidea. We remark that this corresponds with the approach taken by WoRMS [63].

## Conclusion: sequences and proper identification of specimens

A well-known principle in modern systematics is that sequences should be associated with specimens identified with precision, preferably with vouchers deposited in a curated collection [3–5, 12–15, 19, 20, 34, 51]. We previously remarked [3] that this was not the case for the sequences registered in GenBank as *Microcotyle sebastis* for which the paper [49] did not indicate any deposition of material in a collection. We remark that the papers in which complete mitochondrial genomes have been described for eight polyopisthocotylean species often show flaws in the systematics of the sequenced specimens (Table 3). For two species, the specimens were only identified at the genus level; in the remaining six, no specimens were deposited for four species, and specimens were deposited only for two species; photographs were attached to the paper for three of the eight species (Table 3). Our aim here is not so much to criticize these works, but rather to offer an example of what needs to be done in the future.

In this paper, we tried to set the minimum requirements for future molecular studies of monogeneans, with at least a drawing, preferably of the specimen itself which was sequenced, measurements, a brief evaluation of the taxonomic status of the species, and specimens deposited in a curated collection. Cutting a specimen into several parts, using (and therefore destroying) one part for the molecular work and depositing the parts of the body which are important for systematics in a curated collection is certainly the best method. We understand, however, that current limitations in technology make this possible and relatively easy only for large polyopisthocotyleans, which contain enough DNA.
